# Beyond the Prompt: Investigating Retrieval-Based Monitoring in Self-Regulated Learning †

**DOI:** 10.3390/jintelligence13080099

**Published:** 2025-08-06

**Authors:** Mengjiao Wu, Christopher A. Was

**Affiliations:** 1 College of Foreign Languages, Shanghai Maritime University, Shanghai 201306, China; 2 Department of Psychological Sciences, Kent State University, Kent, OH 44240, USA; cwas@kent.edu

**Keywords:** metacognitive monitoring, control, retrieval, self-regulated learning

## Abstract

Metacognitive monitoring plays a crucial role in self-regulated learning, as accurate monitoring enables effective control, which in turn impacts learning outcomes. Most studies on metacognitive monitoring have focused on learners’ monitoring abilities when they are explicitly prompted to monitor. However, in real-world educational settings, learners are more often prompted to control their learning, such as deciding whether to allocate additional time to a learning target. The primary goal of this study was to investigate whether retrieval is engaged when learners are explicitly prompted to control their learning processes by making study decisions. To address this, three experiments were conducted. In Experiment 1, participants (N = 39) studied 70 Swahili–English word pairs in a learning task. Each trial displayed a word pair for 8 s, followed by a distractor task (a two-digit mental addition) and a study decision intervention (choose “Study Again” or “Next”). After learning, participants provided a global judgment of learning (JOL), estimating their overall recall accuracy. Finally, they completed a cued recall test (Swahili cue). Responses were scored for accuracy and analyzed alongside study decisions, study decision reaction time (RT), and metacognitive judgments. Reaction times (RTs) for study decisions correlated positively with test accuracy, global judgments of learning (JOLs), and judgments of confidence (JOCs), suggesting retrieval likely underlies these decisions. Experiment 2 (N = 74, between-subjects) compared memory performance and intervention response time between single-study, restudy, retrieval (explicit recall prompt), and study decision (study decision prompt) groups to have better control over study time and cognitive processes. Although no significant group differences in test accuracy emerged, the retrieval group took longer to respond than the study decision group. Within-subject analyses revealed similar recall accuracy patterns: participants recalled successfully retrieved or “no restudy” items better than failed-retrieval or “restudy” items, implying shared cognitive processes underlying retrieval and study decision interventions. Experiment 3 (N = 74, within-subject, three learning conditions: single-study, retrieval, and study decision) replicated these findings, with no condition effects on test accuracy but longer RT for retrieval than study decisions. The similar recall accuracy patterns between retrieval and study decision interventions further supported shared cognitive processes underlying both tasks. Self-reports across experiments confirmed retrieval engagement in both retrieval and study decision interventions. Collectively, the results suggest that retrieval likely supports study decisions but may occur less frequently or less deeply than under explicit monitoring prompts. Additionally, this study explored objective, online measures to detect retrieval-based metacognitive monitoring.

## 1. Introduction

Metacognition refers to the cognition and control of one’s own cognitive activities, playing a critical role in the development of social learning, cognition, and education (Flavell 1979). It is a key component of self-regulated learning, significantly influencing learning outcomes (Dunlosky and Metcalfe 2008; Koriat 2007; Paris and Winograd 1990). According to Dunlosky and Metcalfe (2008), metacognition consists of three components: metacognitive knowledge, metacognitive monitoring, and metacognitive control. Metacognitive knowledge refers to people’s declarative knowledge about cognition. Metacognitive monitoring involves the evaluation of an ongoing cognitive process (i.e., how well information has been learned and the perceived likelihood of recalling the information in the future), whereas metacognitive control refers to the regulation of cognitive activities (e.g., the content, method, time, and duration of study; Dunlosky and Metcalfe 2008). Both the metacognition (Dunlosky and Metcalfe 2008; Nelson and Narens 1990; Thiede et al. 2003) and self-regulated learning frameworks (Koriat 2007; Nelson and Leonesio 1988; Winne and Hadwin 1998) postulate that monitoring informs control, which thereafter influences learning. It suggests metacognitive learners often monitor their performance and subsequently control their learning (e.g., change learning strategy) based on the monitoring results when needed (Wu and Was 2023). For instance, a student learning to read may monitor their performance, deem it inadequate, and control it by deciding to practice for 30 min longer each day. Therefore, as effective monitoring is the prerequisite of effective control (Rawson et al. 2011), the current study addresses metacognitive monitoring.

Research has demonstrated that metacognitive monitoring can support learning outcomes (Dunlosky et al. 2005; Maki 1998; Zimmerman and Kitsantas 1999). Monitoring prompts (e.g., “How well do you think you have learned the material?” or “How well do you remember the word you just learned?”) can support learning because they prompt the learner to monitor and evaluate their cognitive activities (e.g., make judgments of knowing or judgments of learning), whereas control prompts (e.g., “Do you want to spend more time on the learning material?”) directly prompt learners to regulate their cognitive activities (e.g., make a study decision or change a study strategy). Most existing studies on metacognitive monitoring were conducted using explicit metacognitive monitoring prompts, which means the metacognitive monitoring is performed under external instruction to do so for the sake of monitoring one’s own learning (Dunlosky and Nelson 1992; Nietfeld et al. 2005; Roebers et al. 2014; Schraw and Roedel 1994; Thiede et al. 2003). For example, Thiede et al. (2003) asked participants to rate their comprehension of the text after reading using the query “How well do you think you understood the passage whose title is listed above? 1 (very poorly) to 7 (very well).” Nietfeld et al. (2005) measured global monitoring accuracy by asking participants to rate their confidence in their test performance immediately after they finished the test using “Please indicate how confident you are in your overall performance on this test”. In these cases, learners were explicitly prompted to monitor, to rate their comprehension of the text or to rate their test performance confidence. These studies illustrate the impact of metacognitive monitoring on learning as well as learners’ abilities to monitor their learning. However, these studies are limited such that they may not shed light on learners’ metacognitive monitoring behaviors or abilities when no explicit monitoring prompt is provided.

In fact, in many real-world educational settings, students are more often presented with control prompts rather than monitoring prompts. For instance, a language teacher might ask a student whether additional explanation and examples are necessary for better understanding of a grammar point or whether the student would prefer to practice the new grammar point through more exercises. Such scenarios explicitly prompt the student to engage in control processes, making decisions about whether to seek further explanation or practice, rather than prompt the student to engage in monitoring. However, effective control is contingent upon accurate monitoring (Nelson and Narens 1990; Renner and Renner 2001). A student is more likely to make effective study decisions when those decisions are informed by accurate monitoring.

Even if a learner is capable of effectively monitoring their learning when prompted, it does not necessarily mean they will engage in metacognitive monitoring when those prompts are absent. Poor metacognitive control and low performance may be more attributable to a lack of engagement in metacognitive monitoring than to the learner’s ability to monitor. Furthermore, the extent to which a learner can apply their monitoring abilities without explicit prompts may have a greater impact on learning outcomes compared to situations where such prompts are provided. Therefore, examining learners’ metacognitive monitoring behaviors and performance in the absence of explicit prompts has important theoretical and practical implications for learning and education. Do learners still monitor learning even when they are not prompted to monitor but prompted to control? How often do they monitor? How well can they monitor? What strategies do they use? But little is known regarding learners’ monitoring behavior and performance when they are prompted to control. The current study proposed to investigate whether learners engage in metacognitive monitoring during self-regulated learning when prompted to control (i.e., asked to make study decisions) rather than to monitor.

Judgments of learning (JOLs) are a measure of individuals’ subjective evaluation of their own learning. JOLs can be made based on many criteria, such as target retrievability, feeling of knowing, cue familiarity, task difficulty, etc. Among them, JOLs made based on retrieval results (retrieval-based JOLs; the evaluation of learning based on whether the target content is retrievable) are found to be more accurate than the JOLs made based on other criteria such as cue familiarity (Metcalfe and Finn 2008; Karpicke and Smith 2012). According to Metcalfe and Finn (2008), retrieving the target content is an important strategy in making accurate metacognitive monitoring, and only retrieval-based JOLs could enhance the JOL accuracy.

In fact, retrieval is not only a valuable monitoring strategy but also a highly investigated mnemonic strategy that contributes to better learning and memory performance by strengthening retention (Akdoğan et al. 2016; Karpicke 2009; Karpicke and Roediger 2007; Landauer and Bjork 1978; Rawson and Dunlosky 2011). The underlying retrieval practice improves retention by increasing elaboration of mnemonic representations and adding retrieval paths to the memory (Bjork 1975; Carpenter 2009). When individuals attempt to retrieve target information, their retrieving actions will activate related information in long-term memory. This helps to elaborate and add more memory paths to the target information and facilitate future access to the information, which will result in better retention. Retrieval practice is also reported to improve retention by enhancing the mental organization and idiosyncratic processing of information (Hunt 2006). Furthermore, both successful and failed retrieval during encoding could be beneficial for memory performance either by enhancing memory strength or making less effective mediators stronger in specific learning settings (Bahrick and Hall 2005; Halamish and Bjork 2011; Landauer and Bjork 1978; Pyc and Rawson 2010). Specifically, unsuccessful retrieval, if followed by feedback and restudy opportunities, may also benefit long-term retention (Bjork 1994). Retrieval-based judgment is also believed to be the only judgment that could improve memory performance (Metcalfe and Finn 2008). Therefore, retrieval-based judgments should enhance both learning performance and metacognitive monitoring accuracy.

Thus, this study specifically focused on retrieval-based monitoring, particularly in the absence of explicit monitoring prompts. The primary goal was to determine whether learners spontaneously engage in metacognitive monitoring using a retrieval strategy when prompted to control during a self-regulated learning paradigm, as well as its impact on memory performance and learning. This focus does not imply that retrieval is necessary for metacognitive monitoring, nor that retrieval-based monitoring is the only form deserving research attention. Motivated by the positive impact of retrieval on metacognitive monitoring accuracy and learning performance, this study attempted to gain a deeper understanding of the monitoring that uses retrieval as a monitoring strategy and whether such a strategy is commonly employed during self-regulated learning.

Another characteristic of retrieval is that it consumes a certain amount of time (Staszewski 1988). For example, Metcalfe and Finn (2008) argued that retrieval-based judgments are slower than the familiarity-based judgments because retrieval takes longer. Reder and Ritter (1992) found 850 milliseconds to be the cutoff time for retrieval: if a decision is made within this time frame, it will be impossible for individuals to base their decisions on completed retrievals, as the time is too short to complete retrieving. Thus, if a study decision is made based on retrieval, it will take the individual at least the cutoff time to complete the retrieval process. Then a study decision made within the cutoff time might result in lower learning performance due to the lack of the positive impact of retrieval on both learning and metacognitive monitoring. Moreover, it is important to recognize that the cutoff time of retrieval may vary depending on the nature and complexity of each task.

On the other hand, it is reported that the existing assessments of metacognitive monitoring may have two methodological weaknesses: (1) the dependence on subjective self-report by participants (Nelson and Dunlosky 1991); and (2) the use of offline measures (Bryce et al. 2015). Commonly used measures of metacognitive monitoring are self-reported, in which participants are asked to make subjective judgments of their own cognitive processes (i.e., JOLs ask participants to rate how well they have learned the target materials). This could potentially reduce the reliability of the measurements. Offline measures might not be able to reflect what occurs during the task because they are usually remote from the target cognitive task (i.e., delayed JOLs are made a period after the learning). Thus, objective and online measures of metacognitive monitoring are likely to offer greater reliability by minimizing biases inherent in self-reports and capturing real-time processes. Some researchers have started to apply neuroscientific approaches such as event-related potentials (ERPs) to explore the mechanism underlying metacognitive monitoring. For example, neuroscientific approaches such as event-related potentials (ERPs) could provide online and direct neural indices of whether fluency (indexed by the N400) contributes to JOLs, because ERPs provide an effective means to distinguish between fast, automatic processes like fluency and more deliberate, belief-influenced processes involved in the generation of JOLs (Undorf et al. 2020). ERPs were also used by Müller et al. (2016) to investigate the underlying neural correlates of metacognitive monitoring, JOLs in specific. Cong and Jia (2022) compared the amplitude of ERP components and brain activation areas between high and low ease of learning (EOL) judgments to test the ease-of-processing hypothesis.

Some researchers have started to apply neuroscientific approaches such as event-related potentials (ERPs) to explore the mechanism underlying metacognitive monitoring (Cong and Jia 2022; Müller et al. 2016; Undorf et al. 2020) for more direct and objective evidence. Therefore, the secondary goal of this study was to report the attempts of measuring retrieval-based metacognitive monitoring using online and objective measures, including measuring the response time of study decision-making and test accuracy. It was hypothesized that learners will (1) monitor their learning using retrieval strategies when prompted to control their self-regulated learning; and (2) the retrieval-based metacognitive monitoring of learning is detectable with online and objective measures.

## 2. Experiment 1

Experiment 1 addressed whether learners would monitor their learning processes using retrieval strategies when they were explicitly prompted to control their learning processes. Specifically, it examined whether learners would try to retrieve the learning target when asked to make study decisions between restudy and advancing to the next item. In addition, Experiment 1 investigated the effects of the retrieval-based monitoring on learning outcomes, and whether learners were aware of their retrieval behaviors.

It was inferred that study decisions made based on retrieval results would likely lead to higher recall accuracy in subsequent cued recall tests, due to the benefits of retrieval on memory and learning performance (Karpicke and Roediger 2007; Karpicke 2009; Rawson and Dunlosky 2011). When retrieval is involved in study decision-making, a minimum cutoff time for retrieval is required (Reder and Ritter 1992). If a study decision is made within this cutoff time, retrieval likely has not occurred, potentially resulting in lower test accuracy. Conversely, if the decision is made after the cutoff time, retrieval may have taken place, though this is not guaranteed, as learners could be distracted, guessing, or mind-wandering. However, when retrieval is involved, it can enhance learning performance and improve test accuracy. Additionally, longer study decision response times may indicate greater effort in retrieval, which could lead to higher test accuracy.

For this study, locating the retrieval cutoff time in this experiment setting could indicate a greater likelihood of retrieval.

### 2.1. Hypotheses

**Hypothesis 1.1.** 
*Study decision response time will be positively associated with cued recall test accuracy.*

Study decisions made based on retrieval results are assumed to lead to higher recall accuracy. Retrieval takes time. Thus, it is predicted that there will be a positive correlation between study decision response time and test accuracy.

**Hypothesis 1.2.** 
*The study decision of restudy, which gives learners more study time, will lead to better cued recall test accuracy.*

**Hypothesis 1.3.** 
*The cutoff time of retrieval can be located.*

That is, decisions made under the cutoff time of retrieval will result in lower cued recall test accuracy, whereas decisions made beyond the cutoff time will lead to higher cued recall test accuracy. The location of the cutoff time of retrieval will help support the likelihood of retrieval.

### 2.2. Method

Participants: Participants included thirty-nine (twenty-two females, seventeen males) sophomore- or junior-level college students (mean age = 20.36, *SD* = 1.22) enrolled in an educational psychology course at a public university in Northeast Ohio. Students received course credit for participation. While an a priori power analysis was not conducted, post hoc sensitivity analyses (using G*Power 3.1) indicated that N = 39 provided 80% power to detect correlations of r ≥ 0.42 (α = 0.05, two-tailed), corresponding to a medium-to-large effect size.

**Figure 1:**
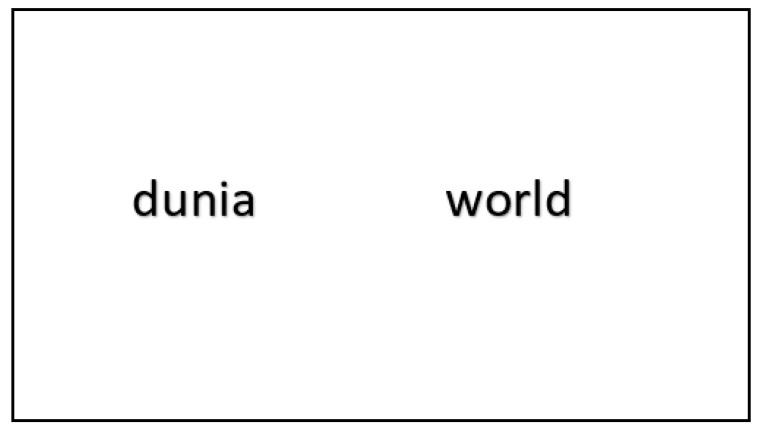
Sample of word pair presentation.

Stimuli: All stimulus presentations were programmed and administered on a computer using the custom program, E-prime 2.0. All participants were assigned to and seated in front of a 15-inch desktop computer, with up to four participants present at the same time. The computers were separated by boards to reduce distraction from the other participants and other computers. The stimuli included the Swahili–English paired associates published by Nelson and Dunlosky (1994), which were normed by difficulty level. The Swahili–English word pairs were used as learning and testing materials in this study (e.g., dunia–world). For each word pair, the Swahili word and a one-word English translation on the right side were presented on the computer screen for a certain amount of time during the learning phase, one word pair at a time. An example of the presentation of a Swahili–English word pair is shown in Figure 1.

**Figure 2:**
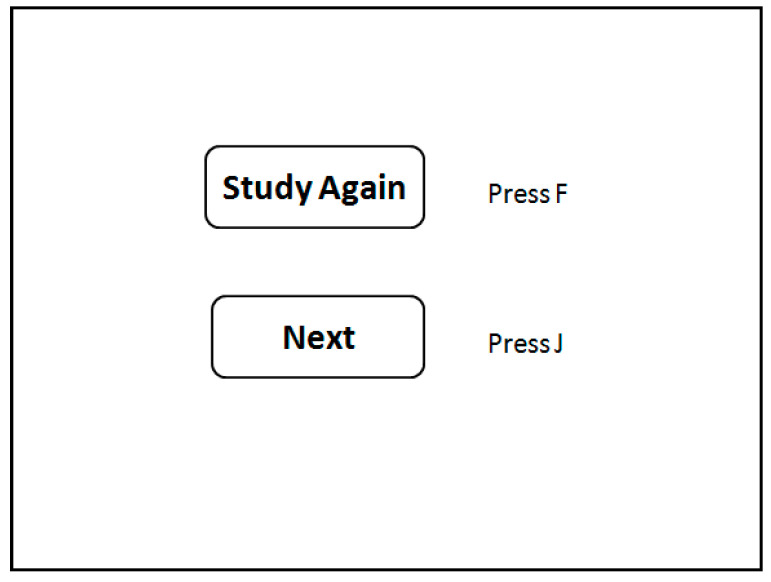
Screen example of the study decision intervention in Experiment 1.

Procedure: At the beginning of the experiment, participants answered some background questions. Then participants were provided a brief overview of the experiment, including being told they would study 70 Swahili–English word pairs in this experiment and that they would be tested in a cued recall test at the end of the experiment. After two practice trials, participants were told that the main task would start. Their task goal was to learn as many word pairs as possible and recall at least 70% of the word pairs in the final test. The main task consisted of a learning phase, a global judgment of learning (a global JOL), and testing. Individual JOLs were avoided because they would serve as explicit prompts for monitoring. A global JOL that asked for the self-evaluation of the overall learning performance of all target items was implemented after the presentations of all items. The learning phase was composed of 70 learning trials. At the beginning of each trial, a blank screen with a black “+” in the middle of the screen was presented for 500 milliseconds to prepare the participants for the appearance of the upcoming item. In each trial, participants were presented with a Swahili–English word pair for 8 s. Then they were instructed to solve a distractor task and make a study decision regarding the next study step. Each item was presented after a 500-millisecond delay, in which a blank screen with a black “+” in the middle of the screen was presented. The presentation of a word pair was immediately followed by a distractor task, which was used to prevent participants from rehearsing the item. The distractor task was a two-digit mental addition task. Participants were instructed to type in the answer to the addition question and press the “Enter” key on the keyboard to submit the answer within 20 s. Once an answer was submitted, the screen indicated whether it was correct or incorrect for 1500 ms and then moved to the study decision intervention. A lack of response within 20 s would be treated as an incorrect response. In the self-paced study decision intervention, participants were presented with two options (“Study Again” and “Next”) vertically placed in the center area of the computer screen (see Figure 2 for the screenshot). Participants were instructed to press the “F” key on the keyboard to see the same item for another 4 s and press the “J” key to advance to the next item. The study decision response time (study decision RT) was the time it took from the onset of the stimuli of the study decision intervention to the time a decision was submitted.

Upon completion of all the learning trials involving the 70 Swahili–English word pairs, participants were prompted to provide a self-paced global judgment of learning (JOL), rating how well they thought they had learned the Swahili–English word pairs, e.g., “You have learned 70 Swahili–English word pairs. How well do you think you have learned? Please rate your overall learning from 0% to 100%.” Participants were instructed to type in their global JOL into a designated area on the screen and use the “Enter” key to submit their responses. After the global JOL, participants answered four survey questions regarding the learning processes to identify whether they were aware of certain cognitive processes (e.g., Have you ever selected the option of “study again”? Did you recall the last pair when selecting “Study again”? Have you ever selected the option of “Next”? Did you recall the last pair when selecting “Next”?). In the end, a cued recall test was used to assess participants’ learning performance of the 70 Swahili–English word pairs. The pairs were presented in random order. In testing, the Swahili word was presented on the left side of the screen (the cue, e.g., dunia –?). Participants needed to recall the English equivalent translation (e.g., world) and type the answer into the designated area on the screen. Participants were instructed to submit their responses by pressing the “Enter” key. A lack of response within 20 s would be treated as an incorrect recall. Each word test was followed by a self-paced judgment of confidence (JOC) in the attempted response (e.g., “*How confident are you in your answer?*”) on a scale ranging from 0 (no confidence) to 100 (complete confidence). Participants were instructed to input their confidence rate into a designated area on the screen and press “Enter” to advance to the next testing item. An item was scored as correct or incorrect by matching the typed response with the correct English target. The typed answers were also manually checked and rescored by researchers after the experiment. If the response and the correct answer matched morphologically (to eliminate typos) or semantically (to accept synonyms), the response was counted as correct. Otherwise, the response was counted as incorrect. After participants completed the final cued recall test, they were thanked and dismissed. All participants completed the assessment within an hour.

### 2.3. Results

In the final cued recall test, every participant was tested on their memory of the 70 Swahili–English word pairs. In the test trials, a correct response was coded as 1, and an incorrect response was coded as 0. Test accuracy, defined as the mean proportion of correct responses on the final cued recall test across all 70 trials, ranged from 0 to 1 for each participant, *M* = 0.16, *SD* = 0.13. The accuracy of the distraction task (mental addition of a two-digit addition) was high with a small range of distribution (*M* = 0.88, *SD* = 0.06), suggesting that participants paid attention to the experiment. A decision to restudy was coded as 0, and the decision to advance to the “next item” was re-coded as 1. Thus, the aggregated study decision responses ranged from 0 to 1, representing the proportion of the decision of advancing to the next item without restudy. The means and standard deviations of the study decision, study decision response time (RT), test accuracy, global JOL, and individual JOC are presented in Table 1.

In order to test H1.1 and H1.2, correlations were run to analyze the relationships among the variables in Experiment 1 (see Table 2): the study decision, study decision RT, test accuracy, global JOL, and individual JOC. Study decision RT was positively correlated to test accuracy (*r*(39) = 0.34, *p* = 0.03), global JOLs (*r*(38) = 0.34, *p* = 0.04), and individual JOC (*r*(39) = 0.37, *p* = 0.02). Thus, H1.1 was supported. Study decision RT was negatively correlated to study decision (*r*(39) = −0.33, *p* = 0.04). The study decision, on the other hand, only correlated with study decision RT but had no correlation with test accuracy or global JOL. Thus, H1.2 was not supported. The test accuracy was positively correlated with global JOLs (r(38) = 0.55, *p* < 0.001) and individual JOCs (r(39) = 0.71, *p* < 0.001). Post hoc power analyses revealed that our sample size (N = 39) provided 99% power to detect the strongest observed correlation (test accuracy with individual JOC, r = 0.71), but only 68% power for medium effects (e.g., study decision RT with test accuracy, r = 0.34).

To understand whether study decision RT and study decision interacted to account for the variance in memory performance, a linear regression was conducted with the aggregated data with centered study decision RT, centered study decision, and their interaction as the independent variable, and the mean test accuracy as the dependent variable. The model was not significant, F(3, 35) = 1.56, *p* = 0.22. The adjusted R^2^ = 0.043. None of the predictors were significant. Thus, there was no interaction between study decision RT and study decision on test accuracy.

To further test H1.1 and H1.2 and examine whether study decision and study decision RT could be used to explain the variance in final test accuracy, a generalized linear mixed model was run on the full data (see Table 3). In the model, item-level study decision and item-level study decision RT were used as independent variables, item accuracy, defined as the binary outcome of each cued recall trial of the cued recall test (“0” for incorrect and “1” for correct), as the dependent variable, and participants and the word pairs as random effects. Study decision RT for each trial was measured in seconds. The model was significant, *F*(2, 2727) = 4.77, *p* = 0.009. Item-level study decision RT was found to account for a significant amount of variance in item accuracy, *F*(1, 2727) = 9.52, *p* = 0.002, CI [0.05, 0.21], but item-level study decision was not a significant predictor.

It was argued that a study decision made within the cutoff time would result in lower learning performance due to the lack of the positive impact of retrieval on both learning and metacognitive monitoring. Therefore, we tried to locate the cutoff time of retrieval in Experiment 1 to test H1.3. We first examined whether 850 ms (Reder and Ritter 1992) was the cutoff time of retrieval in Experiment 1 by comparing participants’ test accuracies of the word pairs they spent over and under 850 ms on making study decisions in the full data. A paired sample *t*-test showed no significant difference in participants’ test accuracies for the word pairs they spent over or under 850 ms on making the study decisions. This indicated 850 ms was not the cutoff time of retrieval in Experiment 1. A possible explanation was that the current experiment required a different motor reaction from Reder and Ritter’s (1992) experiment, which resulted in a different cutoff time that could be collected. In order to locate the cutoff time for Experiment 1, a regression analysis was run on the full data with the item accuracy in the cued recall test used as the independent variable and study decision RT as the dependent variable. The item accuracy was coded as 1 = correct and 0 = incorrect. Study decision RT was measured in milliseconds. The model was significant, *F*(1, 2728) = 30.62, *p* < 0.001, *R*^2^ = 0.01. The regression equation was study decision RT = 1058.60 + 367.80 × (item accuracy); see the regression statistics in Table 4. The observed average study decision RT increased by 367.80 ms for a correct response. The values 1058.60 ms and 1426.40 ms are the expected values for incorrectly and correctly answered items. Three paired sample *t*-tests were run, respectively, using 1058.60 ms, 1426.40 ms, and their mean of 1242.5 ms as the cutoff time to examine which one suited the data better. The mean test accuracy was significantly higher for the pairs with study decisions RT over 1242.5 ms (*n* = 39, *M* = 0.21, *SD* = 0.21) than for the pairs with the study decisions RT under 1242.5 ms (*n* = 39, *M* = 0.15, *SD* = 0.13), *t*(38) = 2.15, *p* = 0.04; the mean test accuracy was significantly higher for the pairs with study decisions RT over 1426.4 ms (*n* = 39, *M* = 0.22, *SD* = 0.03) than for the pairs with the study decisions RT under 1426.4 ms (*n* = 39, *M* = 0.15, *SD* = 0.02), *t*(38) = 2.35, *p* = 0.02, showing a significant impact of study decision RT. No significant difference was found in participants’ mean test accuracy between the pairs with study decisions RT over and under 1058.6 ms. The results suggested that when participants spent more than 1242.5 ms (including 1426.4 ms) on making study decisions, they were more likely to gain a higher proportion of correct responses in the test. Thus, 1242.5 ms was more likely to be the valid retrieval cutoff of study decision RT in Experiment 1.

This experiment also examined whether participants were aware of their retrieval behaviors in self-regulated learning. After the global JOL but before the cued recall test, participants were asked whether they had ever chosen to restudy, and if they had, if they did try to recall the word pair before making the decision. Thirty out of thirty-nine participants claimed that they had chosen to restudy, and 57% of these thirty participants (seventeen participants) who claimed to have chosen to restudy stated they “tried to recall the word pair before making the decision of restudy”. The mean study decision RT was 1381.40 ms (SD = 689.03) for the participants who claimed to have recalled the word pair before making the restudy decision. The mean study decision RT was 827.69 ms (SD = 386.70) for the participants who claimed that they did not recall the word pair before making the restudy decision. The independent sample *t*-test analysis showed that participants who claimed that they recalled the word pair before making the restudy decision spent significantly more time on making that decision than the ones that claimed to have made no recall, t(28) = 2.60, *p* = 0.02, 95% CI [116.64, 990.78]. The participants were also asked whether they had ever chosen to move on to the next pair without restudy, and if they had, whether they had tried to recall the word pair before making that decision. Thirty-five out of the thirty-nine participants claimed they had selected to move on to the next pair directly, 71% of whom (twenty-five participants) stated they tried to recall the word pair before making the decision. Another independent sample *t*-test was calculated to compare whether participants who claimed to have recalled the item before deciding to advance to the next item spent more time on making that decision than the participants who claimed to have made no recall. The results showed that participants who claimed to have recalled the item before deciding to have no restudy spent significantly more time (M = 1315.45 ms, SD = 612.11) on making the decision than the participants that claimed to have performed no recall (M = 811.41 ms, SD = 418.75), t(33) = 2.38, *p* = 0.02, 95% CI [73.20, 934.88]. Thus, participants that reported retrieval during decision-making spent significantly more time on making decisions than the ones that reported no retrieval, which indicated that participants were aware of their retrieval behaviors during study decision-making.

### 2.4. Discussion

The correlation results showed that study decision RT was negatively correlated with study decision but positively associated with test accuracy. That means when participants spent more time (RT) on making study decisions, they were more likely to choose to restudy, and they were more likely to have a higher recall test accuracy. The results in the linear mixed model also showed that study decision RT could account for a significant amount of variance in the item accuracy in the cued recall test. Taken together, the results supported H1.1, indicating that study decision RT was positively associated with test accuracy (both at the mean level and item level) and highlighted the important impact of study decision RT on the later retention, suggesting the importance of the cognitive activity during study decision-making.

Why did the study decision RT matter? What were participants doing when making study decisions? Why would they remember better after spending more time on making study decisions? They might be guessing or mind-wandering. But guessing or mind-wandering would not enhance memory performance. The time participants spent on making the study decisions was meaningful. Since retrieval consumes time, the positive impact of study decision RT on test accuracy postulates that the mechanism underlying study decision-making may be an attempted retrieval of the targets. It is thus argued that in Experiment 1, it was the attempted retrieval that took extra time and led to better accuracy in the later retention.

Experiment 1 found 1242.5 ms to be the cutoff time of retrieval during the study decision-making. It is longer than the motor reaction time reported in Reder and Ritter’s (1992) study. In their experiment, participants were presented with a math calculation problem and had to choose between retrieving or calculating the answer by pressing one of two buttons. Critically, participants had their fingers ready on the buttons to generate the fastest motor reaction. In contrast, the current experiment asked participants to make the study decisions by pressing the key “F” or key “J” on a standard 84-key PC keyboard. Importantly, right before the study decision intervention, participants were instructed to solve a two-digit math addition. To submit the answer to the math question, participants needed to submit their answers by typing in the number and pressing the key “Enter”. This required them to move their fingers from “Enter” to either “F” or “J” to make their subsequent study decision, inevitably increasing motor reaction time. Given these procedural differences, particularly the additional finger movement required, a longer retrieval cutoff time in the present study (compared to Reder and Ritter’s 850 ms) is both expected and justified.

The location of the cutoff time in the study decision RT further indicates a possible occurrence of retrieval during the process of study decision-making in Experiment 1. It implies when asked to make study decisions, participants are very likely to base their study decisions on retrieval results. This indicates the possibility that learners spontaneously monitor their learning processes using a retrieval strategy when prompted to control (make study decisions) in self-regulated learning.

It was also found that participants who reported retrieval behaviors during decision-making spent significantly more time on making decisions than the participants that claimed to practice no retrieval, suggesting that participants were aware of their retrieval behaviors during study decision-making. However, there are limitations in these analyses, and they should be interpreted with caution. First, the grouping of participants was based on self-reported retrieval behaviors, which could introduce potential confounding variables (e.g., individual differences in metacognitive awareness or response bias). Additionally, the relatively small sample sizes may reduce statistical power and increase the risk of Type I or II errors. The uneven subgroup sizes (e.g., 25 vs. 10 participants in the second independent sample *t*-test analysis) could introduce additional variability in response time. Despite these limitations, the consistency in results (longer decision times when retrieval was reported) across both restudy and “no restudy” decisions suggests that participants’ self-reports align with behavioral measures, supporting the conclusion that retrieval attempts were most likely conscious during decision-making. Future research with larger samples and controlled retrieval conditions could further validate these findings.

On the other hand, no significant association was found between study decisions and test accuracy, despite the fact that choosing to restudy increased study frequency and duration. Consequently, H1.2, which posited that restudy could enhance learning performance, was not supported. This null effect may be attributable to a floor effect, given the low mean test accuracy (M = 0.16, SD = 0.13, N = 39). To address this, Experiment 2 re-examined the impact of extended study time by reducing task difficulty, specifically, by reducing the number of target learning items, to test whether the floor effect could be mitigated. The low test accuracy in Experiment 1 likely reflected high task difficulty rather than participant disengagement. The Swahili–English word pairs were derived from Nelson and Dunlosky (1994)’s word list, which reported normed recall performance after multiple learning sessions. In their study, the mean recall accuracy after the first learning of 50 word pairs (10 s per pair) was similarly low (M = 0.12). Moreover, participants’ high distractor task accuracy (M = 0.88, SD = 0.06) in Experiment 1 confirmed their sustained attention and effort, ruling out alternative explanations such as random responding, low motivation, or strategic task avoidance.

Although our study was well-powered to detect medium-to-large effects (r ≥ 0.35), weaker correlations (e.g., r < 0.30) may have been undetected due to limited sample size. Future research with larger samples is needed to explore these smaller effects.

In conclusion, the findings revealed a positive association between the time participants spent on making study decisions and their memory performance. Combined with the location of the cutoff time for retrieval, these results suggest that retrieval is likely occurring during the process of study decision-making, thereby contributing to improved memory performance. It is also likely that learners are consciously aware of their retrieval behaviors. However, while study decisions influence study time and frequency, they have no significant impact on subsequent retention in the current learning context.

## 3. Experiment 2

Two key findings were drawn from Experiment 1. First, the more time learners spent on making study decisions, the more likely they would achieve better retention, suggesting retrieval might play a role in cognitive processing when participants make study decisions in self-regulated learning. Second, increased study time and frequency (restudy) did not enhance retention in the current learning context. To assess the generalizability of these findings and to continue addressing the overarching question of whether retrieval is practiced during study decision-making when learners are explicitly prompted to control their learning processes, Experiment 2 was designed with improved control over study time and the underlying cognitive processes using a between-subject design. Participants were randomly assigned to one of four learning settings: Single-study group: Items were presented for 8 s (one exposure), followed by a distractor task. Restudy group: Items were presented for 8 s, followed by a distractor task, and then presented again for 4 s (two exposures). Retrieval group: Items were presented for 8 s, followed by a distraction task and then a retrieval prompt, where participants attempted to recall and report the item. Study decision group: Items were presented for 8 s, followed by a distraction task and then a control prompt, where participants chose to either restudy the item or move on to the next one. This design allowed us to directly compare the single-study and restudy groups to test whether increased study time (from 8 s to 12 s total) and additional exposure would enhance retention. The retrieval and study decision groups served to answer the second research question: Do learners exhibit similar behavior patterns (intervention reaction time and performance) when asked to retrieve information as they do when making study decisions?

### 3.1. Method

Participants: Seventy-four participants (forty-six females, thirty males; mean age = 20.74, *SD* = 1.52) took part in the experiment for course credit. They were at the sophomore or junior level and recruited from college students enrolled in educational psychology at a public university in Northeast Ohio. None of them had participated in Experiment 1.

Stimuli: The same stimuli as in Experiment 1 were used in Experiment 2. Due to the low average test accuracy in Experiment 1 (*M* = 0.16, *SD* = 0.13), the number of target word pairs was reduced from 70 to 50 in Experiment 2.

Procedure: Participants were randomly assigned into four groups: a single-study group, a restudy group, a retrieval group, and a study decision group. The experimental procedures of all four groups in Experiment 2 were like that in Experiment 1 but distinguished each other in terms of the study time and interventions. All four groups were instructed to study 50 Swahili–English word pairs. The presentation of each item was immediately followed by the same distraction task, a two-digit mental addition task used in Experiment 1. The single-study group was presented with each item once for 8 s, followed by the same distraction task with no intervention (one exposure, no intervention prompt). The restudy group was presented with each item first for 8 s, followed by the distraction task, and then was presented with the same item for another 4 s (12 s in total; two exposures, no intervention prompt). The retrieval group was presented with each item for 8 s, followed by the distraction task, and then was instructed to retrieve the item and report the retrieval results (one exposure, retrieval prompt). In the retrieval intervention, participants saw a prompt question, “Now try to retrieve the last pair. Do you remember it?” Two alternatives, “Yes” and “No”, were presented in the lower 1/3 of the screen as answer options. Participants reported their retrieval results by pressing the key “F” for yes or the key “J” for no. Key presses other than “F” or “J” would not be accepted. Once an answer was submitted, the program would present a blank screen with a black “+” in the middle of the screen for 500 milliseconds to prepare the participants for the next learning item. The study decision group, after being presented with the item for 8 s and completing the distraction task, was instructed to make a study decision between restudy and advancing to the next item (one exposure, control prompt). In the study decision intervention, the participants saw two options, “Study Again” and “Next”, labeled in two buttons and presented horizontally in the lower 1/3 portion of the screen. The option of “Study Again” referred to seeing the same item for another 4 s, and the option of “Next” meant advancing to the next item immediately. Participants were instructed to press the key “F” for restudy and the key “J” for advancing to the next item. Participants in the study decision group were informed that no additional study time would be provided regardless of the study decision. But participants were encouraged to make an honest study decision. This setting was to make sure the retrieval and study decision group were exposed to the learning target for the same amount of time and participants would not avoid selecting “restudy” to shorten the experiment time. Thus, the only difference that could differentiate the behavior patterns and learning results between these two groups (the retrieval and restudy groups) would be the different interventions (retrieval and study decision interventions). Both interventions were self-paced. After the sequential presentation of all 50 items, participants rated their overall judgments of learning, and their learning performance was tested in a cued recall test.

### 3.2. Assumptions of the Underlying Processes

Our assumptions of the cognitive activities underlying the responses during the retrieval and study decision interventions are presented in Table 5. When a participant in the retrieval group reported that they still remembered the Swahili–English word pair in the retrieval intervention, it was assumed that they attempted to retrieve and successfully retrieved the target item (+ +). When a participant reported that they did not remember the pair, it was assumed that they attempted to recall but failed to retrieve the word pair (+ −). Based on the findings from Experiment 1, we assumed that in an ideal condition, responsible learners would use retrieval results to guide their study decisions when prompted to make a study decision. Therefore, we made the following assumptions accordingly: When a participant chose to restudy, it was because they attempted to retrieve the target information but failed (+ −). When a participant chose to advance to the next item immediately, two possible causes were assumed: They attempted to retrieve and succeeded (+ +), or they did not even attempt to retrieve the item (− N/A). Thus, the choices of “not remember” and “restudy” are both assumed to have resulted from a failed retrieval. The choice of “remember” resulted from a successful retrieval, while the choice of “next” could stem from either a successful retrieval or no retrieval at all, as a convenient way to end the study session. Therefore, we could compare the behavior patterns (intervention reaction time and memory performance) between the retrieval and study decision groups to better understand the cognitive processes in making study decisions. For example, since a retrieval-based judgment would take more time than a judgment based on familiarity

### 3.3. Hypotheses

**Hypothesis 2.1.** 
*The retrieval group and study decision group will achieve similar cued recall test accuracies but higher than the single-study group and restudy group.*

Retrieval is believed to be involved in the study decision-making process; thus, it is assumed that both the study decision and retrieval groups will benefit from the benefits of retrieval on memory.

**Hypothesis 2.2.** 
*The restudy group, which receives more study time and frequency, will perform better than the single-study group in the cued recall test.*

This is a re-examination of the unexpected finding from Experiment 1.

**Hypothesis 2.3.** 
*The time required for making study decisions will be comparable to that of reporting retrieval results.*

This prediction is based on the assumption that both tasks engage retrieval processes. That is, when making study decisions, participants will spontaneously retrieve item information, similar to reporting retrieval results, resulting in equivalent response times between study decision and retrieval interventions.

### 3.4. Results

Descriptive statistics analyses and a one-way analysis of variance (ANOVA) were run on the final test accuracies and global JOLs of all four groups to test H2.1 and H2.2. The means, standard deviations, skewness, and kurtosis of the test accuracies and global JOLs are presented in Table 6. An ANOVA analysis showed no significant between-subject effect on final test accuracies, F(3, 70) = 0.49, *p* = 0.69, *η*^2^ = 0.02, even though the retrieval group had numerically the highest proportion of correct recalls among the four groups. Post hoc power analysis (α = 0.05, f = 0.14) indicated the observed power was low (15%), suggesting the sample size was insufficient to reliably detect the small effect size. Another one-way ANOVA on mean global JOLs showed no significant between-group differences, F(3, 70) = 1.59, *p* = 0.20, *η*^2^ = 0.06, even if the retrieval group had the numerically highest global JOLs, followed by the study decision group. Post hoc analysis (α = 0.05) revealed this analysis had 41% power to detect the observed medium effect size (f = 0.26).

An independent sample *t*-test was run on the retrieval and study decision response time to test H2.3. The mean response time for the retrieval group to report the retrieval results was 2980.88 ms (SD = 1208.46 ms). The mean response time the study decision group used to make study decisions was 1594.49 ms (*SD* = 458.61 ms). The result showed it took the study decision group significantly less time to make the study decision than it took the retrieval group to report the retrieval results, *t*(32) = 4.42, *p* = 0.001, CI [747.84, 2024.95]. The significant difference in response times (d = 1.49) had 99% power (post hoc). This indicates robust sensitivity to detect the observed large effect.

**Figure 3:**
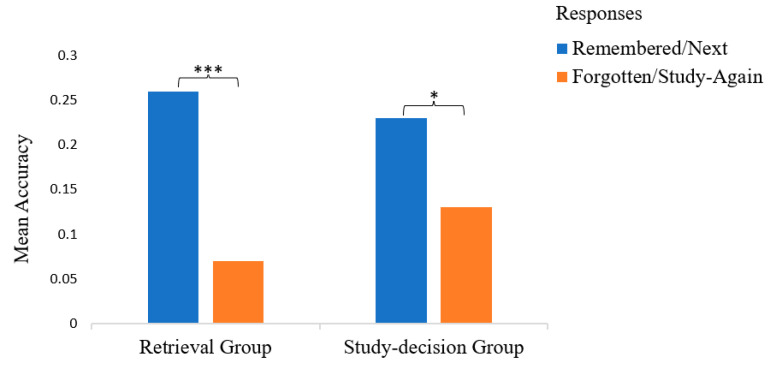
Within-subject effect of intervention response on cued recall test accuracies in the retrieval group and study-decision group in Experiment 2. * *p* < 0.05; *** *p* < 0.001.

Interesting within-subject differences were revealed when comparing the final test accuracies and intervention response time by intervention response in the retrieval group and study decision group. The paired sample *t*-tests results showed that the mean test accuracy for the “remembered” items was 0.26 (*SD* = 0.18), but the test accuracy of the “forgotten” items dropped significantly to 0.07 (*SD* = 0.08), *t*(16) = −10.87, *p* < 0.001). The mean test accuracy of the no-restudy items was 0.23 (*SD* = 0.19), which was significantly higher than the final test accuracy of the restudy item (*M* = 0.13, *SD* = 0.20), *t*(11) = 2.396, *p* = 0.036. The within-subject differences in the final test accuracies by response were significant in both the retrieval group and study decision group; see Figure 3.

**Figure 4:**
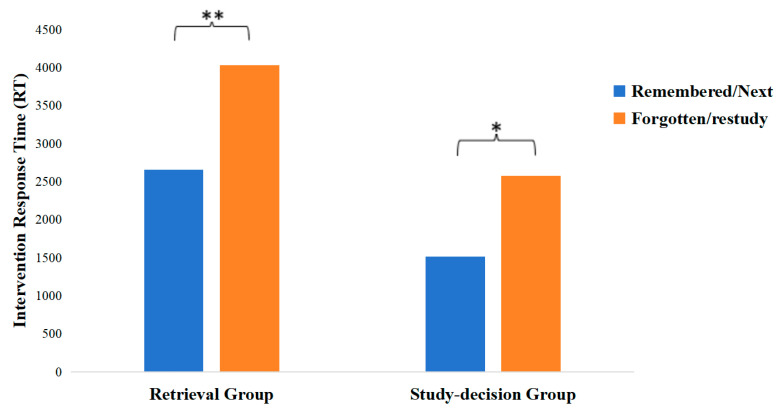
Within-subject effect of response on intervention response time in the retrieval and study-decision groups in Experiment 2. * *p* < 0.05; ** *p* < 0.01.

Another within-subject analysis was run on the intervention RT by response in the retrieval group and study decision group; see Figure 4. A paired sample *t*-test showed participants spent significantly more time on reporting failed retrieval (M = 4029.31 ms, SD = 2276.24) than on reporting successful retrieval (M = 2652.87 ms, SD = 1028.55), t(15) = −3.27, p = 0.005. Another paired sample *t*-test revealed the restudy decision (M = 2574.56 ms, SD = 1024.15) took more time than the no-restudy decision (M = 1518.18 ms, SD = 478.96), t(11) = −2.87, p = 0.015.

### 3.5. Discussion

The retrieval group achieved the best test performance numerically but not statistically. No significant group difference in test accuracy was found between the four groups. Thus, H2.1 and H2.2 were not well supported. The retrieval group took significantly longer time to report retrieval results than the study decision group did to make study decisions. Thus, H2.3 was not supported either.

Although no between-subject effect was found in the test accuracy, within-subject effects were observed in both the retrieval and study decision groups. In the retrieval group, participants were more likely to recall items they previously reported as remembered compared to those they had reported as forgotten. Similarly, in the study decision group, participants were more likely to recall items they had chosen not to restudy over those they had chosen to restudy. The retrieval group also spent significantly more time reporting failed retrievals than successful ones, while the study decision group took longer to make study decisions of restudy compared to decisions of no-restudy. These findings align with our assumptions about the cognitive processes underlying study decision-making: participants opted to restudy pairs they struggled to retrieve and chose not to restudy pairs they successfully retrieved during study decision-making. This suggests that participants did not make random study decisions. The similar patterns in recall results and response times between the retrieval and study decision groups suggest similar cognitive processes underlying the retrieval reporting and study decision-making. Therefore, there is a strong likelihood that participants based their study decisions on retrieval outcomes. This inference indicates that retrieval is likely practiced as a monitoring strategy when learners are explicitly prompted to make study decisions.

One possible explanation that retrieval took a longer time than study decision-making is that retrieval, if practiced at all, was practiced less frequently or at a shallower level when participants were asked to make study decisions compared to when they were asked to report retrieval results. This suggests that retrieval may not have been practiced every time a study decision was made. Another possibility is that when a study decision was required, participants might have initiated the retrieval process but stopped once they sensed the feeling of familiarity and based their study decisions on a feeling of knowing (Dunlosky and Metcalfe 2008) rather than on actual recall. In such cases, participants could make quicker decisions, albeit at a shallower cognitive level.

In Experiment 1, the study decision, which was anticipated to influence learning performance by affecting study time and frequency, unexpectedly showed no significant impact on memory performance. This unexpected result led us to re-investigate the impact in Experiment 2 with a more rigorously controlled between-subject design: the single-study group was allocated 8 s of study time, while the restudy group received 4 more seconds after the first 8 s of learning, with a total of 12 s of study time. The results of Experiment 2 replicated those of Experiment 1, as there was no difference in test accuracy between the single-study and restudy groups. This indicates that increased study time of 4 s and frequency did not enhance learning outcomes within the current context. Although these findings were surprising, they could still be consistent with existing literature on the effect of study time on learning outcomes in both self-paced and experimenter-paced conditions. For instance, in all three experiments by Nelson and Leonesio (1988), the accuracy-emphasis group, despite spending significantly more time on self-paced study, showed no improvement in recall compared to the speed-emphasis group. Similarly, Zimmerman (1975) found no recall advantage in the two-presentation group over the single-presentation group, even though the former spent considerably more time on self-paced study. This phenomenon, often referred to as the “labor-in-vain” effect (Nelson and Leonesio 1988), suggests that additional study time may yield little to no improvement in subsequent recall, possibly due to the attenuation-of-attention effect, where learners may pay less attention during repeated presentations. Given that study time and frequency were not the primary focus of this research, there was no need to further examine their effects in the following experiments.

The overall judgments of learning (global JOLs) were used to measure how well participants thought they learned. By comparing the global JOLs across groups, we examined whether different learning settings and interventions could have an impact on learners’ confidence in their learning performance. Numerically, the retrieval group exhibited the highest global JOLs (M = 45.06%), followed by the study decision group (M = 39.44%), while the single-study (M = 31.95%) and restudy groups (M = 32.82%) reported lower confidence in their learning. Although these differences were not statistically significant, the pattern suggests that participants perceived stronger learning after retrieval practice compared to learning without intervention (single study or restudy). Moreover, the study decision group’s global JOLs intermediated between retrieval and single-study/restudy, suggesting the cognitive process underlying study decision-making also enhanced learners’ confidence in their learning performance. The elevated global JOLs observed in the retrieval group likely reflect the well-documented metacognitive benefits of retrieval practice (Karpicke 2009; Kubik et al. 2022; Mitchum et al. 2016; Yang et al. 2017a; Yang et al. 2017b), which could also be the cognitive mechanism that increased the study decision group’s confidence in their learning, though to a lesser degree than explicit retrieval. For example, retrieval practice could have enhanced sensitivity to mnemonic cues such as ease of learning and retrieval fluency, which boosted participants’ overall judgment of their learning.

In conclusion, no between-subject effects were found between retrieval and study decision groups in test accuracy. The within-subject effects of reaction time and memory performance in those two groups suggested the study decision group very likely went through similar underlying cognitive processes during the intervention as the retrieval group, supporting the assumption that retrieval is practiced as a monitoring strategy during study decision-making. However, the retrieval group took longer to report retrieval outcomes than the study decision group took to make decisions, suggesting that retrieval may have been practiced less frequently or at a shallower level when making study decisions.

There are also limitations, as the non-significant accuracy results (F(3, 70) = 0.49, *p* = 0.69, f = 0.1) had low power (14–15%) to detect the observed small effects. With our sample size, we could only reliably detect larger effects (f > 0.40). Future studies with approximately 480 participants would be needed to properly evaluate whether these small effects are meaningful.

## 4. Experiment 3

Experiment 2 replicated the main findings from Experiment 1 that retrieval is very likely the underlying cognitive process during study decision-making. Experiment 2 further found that retrieval is possibly practiced but at a lower frequency and shallower level when learners are cued to control their learning than when learners are cued to retrieve. Given the findings from Experiments 1 and 2, the third experiment further investigated the intervention reaction time and effects of retrieval-based metacognitive monitoring when learners are explicitly prompted to control their learning using a within-subject design, in which each participant experienced three learning conditions: single-study, retrieval, and study decision. A within-subject design was used to reduce the effect of individual differences and increase the statistical power such that it would better reveal the impact of the prompts (retrieval cues and control prompts) on learning performance. The effect of extended study time in the current learning context—an additional 4 s—was re-examined and confirmed in Experiment 2, eliminating the need to include it in Experiment 3.

**Hypothesis 3.1.** 
*Participants will receive the highest test accuracy in the retrieval learning condition, followed by the study decision condition, then the single-study condition, replicating the key finding from Experiment 2.*

**Hypothesis 3.2.** 
*Reporting retrieval results will take more time than making study decisions, replicating the key finding from Experiment 2.*

This will provide further support for the claim that retrieval is only partially or shallowly involved in the study decision process.

**Hypothesis 3.3.** 
*Participants will better recall the items that were reported as remembered than the ones reported as forgotten, replicating the key finding from Experiment 2.*

**Hypothesis 3.4.** 
*Participants will better recall the “no-restudy” items than the “restudy” items, replicating the key finding from Experiment 2.*

H3.3 and H3.4 will together provide additional evidence that study decisions are not made randomly but rather reflect reliance on retrieval-based mechanisms as in retrieval intervention.

### 4.1. Method

Participants: Seventy-four participants (forty-five females, twenty-nine males; mean age = 21.03, *SD* = 1.31) were recruited among college students in an undergraduate educational psychology course in a public university in Northeast Ohio. Students received course credit for participation. All participants were at the sophomore or junior level at the test time. None of them participated in Experiment 1 or 2.

Stimuli: The same stimuli as in Experiment 1 and Experiment 2 were used in Experiment 3. The number of the Swahili–English word pairs was reduced to 20 in each of the three conditions to increase overall test accuracy. Thus, each participant learned 60 Swahili–English word pairs (20 items * three conditions) in Experiment 3. The difficulty levels of all conditions were balanced according to the difficulty levels of Swahili–English paired associates published by Nelson and Dunlosky (1994).

Procedure: All participants underwent three different learning conditions—single-study condition, retrieval condition, and study decision condition. Once participants completed one condition, they would take a break for 20 s before moving on to the next condition. The order of the conditions was randomized for each participant.

At the beginning of the experiment, participants were presented with a general introduction of the experiment on the computer screen. The introduction was followed by a practice trial, which prepared the participants for the procedure and requirements of the main task in each condition. After the practice trial, participants would see a learning goal. The same learning goal was set for all three conditions: to recall at least 70% of the Swahili–English word pairs in the cued recall test.

After reading the learning goal, participants started the learning phase with three learning blocks. The learning task was the same in each block: to learn 20 Swahili–English word pairs. The items in each block were presented in a randomized order. Each block was differentiated by the learning conditions.

In the single-study condition, Swahili–English word pairs were presented sequentially, with each item displayed for 8 s. A two-digit mental addition distractor task—the same task used in Experiments 1 and 2—was interleaved between items, but no additional prompts were provided. In the retrieval condition, participants viewed each item for 8 s before completing the distractor task. Following this, they were asked to recall the previously presented item and report whether they still remembered it (retrieval prompt). In the study decision condition, after viewing an item for 8 s and completing the distractor task, participants were instructed to decide whether to restudy the item or advance to the next one (control prompt). However, they were informed that no additional study time would be provided regardless of their decision. All interventions were self-paced, and the amount of written instruction was balanced between the retrieval and study decision intervention. At the end of each learning condition, participants rated their overall learning performance (global JOL) on a scale from 0% to 100% and completed a cued recall test, identical to the one used in Experiments 1 and 2.

Once participants completed the learning and testing in all three conditions, they were asked to report whether they attempted retrieval when submitting the responses in each condition. After that, participants were thanked and dismissed.

### 4.2. Results

Two repeated measures ANOVA compared the effect of learning interventions on test accuracies and global JOLs after learning in the single-study, retrieval, and study decision conditions (see descriptive statistics of test accuracies and global JOLs in Table 7) to test H3.1. There were no significant condition effects on final test accuracies, *F*(2, 146) = 0.82, *MSe* = 0.02, *p* = 0.44, *η*^2^ = 0.01.^1^ Post hoc analysis indicated only 23% power to detect a small effect (f = 0.10), suggesting the null result may reflect limited sensitivity. Detecting *η*^2^ = 0.01 with 80% power would require a sample size of at least 500. The mean accuracy of the distractor task in all conditions was 0.90 (*SD* = 0.11), suggesting that participants attended to the task. The repeated measure ANOVA revealed significant condition effects on global JOLs, *F*(2, 122) = 11.99, *MSe* = 210.70, *p* < 0.001, *η*^2^ = 0.16. The post hoc analysis showed, for global JOLs, the RM-ANOVA detected a large effect (power = 99%), confirming robust sensitivity to condition differences. Specifically, the global JOL in the retrieval condition was significantly higher than that in the single-study condition (*F*(1, 61) = 17.98, *p* < 0.001) and higher than that in the study decision condition (*F*(1, 61) = 14.02, *p* < 0.001). No difference was found between the global JOLs in the single-study and study decision conditions.

In Experiment 3, it took participants 2496.22 ms (SD = 770.10) on average to report their retrieval results when learning in the retrieval condition, and it took participants an average of 2248.51 ms (SD = 868.83) to make a study decision when learning in the study decision condition. A paired sample *t*-test was performed to test H3.2. The result showed a significant difference in the intervention response time between the retrieval and study decision conditions, t(73) = −2.71, *p* < 0.01, indicating that participants spent more time on reporting the retrieval results than on making study decisions, supporting H3.2.

**Figure 5:**
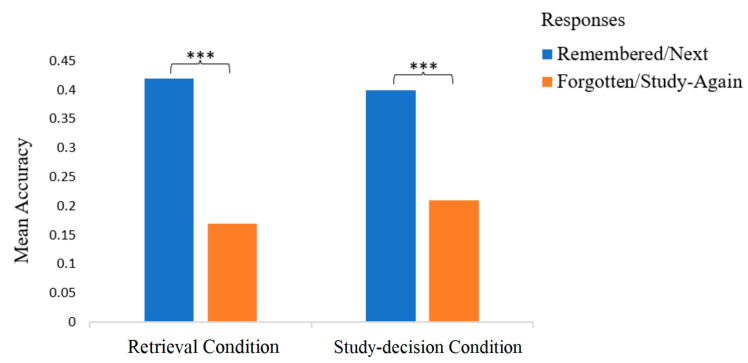
Test accuracies by intervention response in the retrieval and study decision conditions in Experiment 3. *** *p* < 0.001.

A paired sample *t*-test was conducted to test H3.3, revealing significant differences in test accuracies by intervention response (see Figure 5): participants achieved higher recall test accuracy with the items that they reported as successfully retrieved (*M* = 0.42, *SD* = 0.24) than the items that they reported as failed to retrieve (*M* = 0.17, *SD* = 0.29) in retrieval intervention, *t*(59) = 6.92, *p* < 0.001. The recall accuracy was higher for the “remembered” items. Another paired sample *t*-test was conducted to test H3.4, examining recall accuracy as a function of study decision. Participants who made the same study decision on all trials (*n =* 24) were excluded from the analysis. Among the remaining participants, recall accuracy differed significantly between word pairs that were chosen to restudy (*M* = 0.21, *SD* = 0.25) and those not chosen for restudy (*M* = 0.40, *SD* = 0.26), *t*(49) = 4.79, *p* < 0.001. The recall accuracy was higher for the no-restudy items.

Another paired-samples *t*-test was conducted to examine reaction time (RT) differences in the retrieval and study decision interventions based on participants’ responses. Specifically, the analysis assessed whether participants spent different amounts of time making one type of response compared to the other in each condition. After excluding participants who reported the same retrieval outcome for every trial, a significant difference in intervention RT was observed between responses in the retrieval condition. Participants took significantly longer to report unsuccessful retrieval (M = 3909.05 ms, SD = 2989.22 ms, n = 60) than successful retrieval (M = 2442.97 ms, SD = 783.88 ms, n = 60), t(59) = −3.74, *p* < 0.001.

In contrast, no significant difference was found in intervention RT between restudy decisions (M = 2588.18 ms, SD = 965.21 ms, n = 50) and no-restudy decisions (M = 2428.72 ms, SD = 862.24 ms, n = 50) in the study decision condition.

According to the post-learning survey, 74.32% of participants (*n =* 74) reported that they rehearsed and retrieved previously learned word pairs after encountering a new word pair in the single-study condition; additionally, 84.72% (*n =* 72) indicated that they retrieved word pairs when asked to report retrieval results, while 82.19% (*n =* 73) reported engaging in retrieval when making study decisions. Paired sample *t*-tests showed no significant difference between the proportions of self-reported retrieval across conditions.

### 4.3. Discussion

The results of Experiment 3 replicated most of the findings from Experiment 2. No significant differences in test accuracy were observed across learning conditions, even after test scores were improved by reducing the number of learning items. These findings aligned with the between-subject analysis in Experiment 2, where no differences in test accuracy were found among the single-study, re-study, retrieval, and study decision groups. H3.1 was not supported.

One possible explanation for the current findings is that participants may have spontaneously rehearsed and retrieved the target word pairs during and after the initial presentation across all conditions. This spontaneous retrieval could have mitigated the effect of prompts, leading to comparable learning outcomes across conditions. Supporting this explanation, the survey results on self-reported retrieval practice indicated that most participants engaged in retrieval regardless of condition or prompts. This further suggests that retrieval practice occurs spontaneously in self-regulated learning, even in the absence of external prompts.

The significant difference in later recall accuracy across responses in the retrieval and study decision conditions supported the assumption that decisions were not made randomly but were based on retrieval outcomes. Participants better recalled the items that were successfully retrieved compared to those that were not in the retrieval condition. Similarly, participants better recalled the items they chose *not* to restudy than those they opted to restudy. These findings align with those of Experiment 2 and further confirm the assumed underlying cognitive processes involved in study decision-making. That is, restudy decisions (“study again”) are very likely driven by unsuccessful retrieval attempts, whereas non-restudy decisions (“next”) likely resulted from either successful retrieval or no retrieval attempt (as shown in Table 4). These findings support the idea that retrieval-based monitoring is very likely employed when learners are prompted to control their learning by making study decisions. Additionally, a significant within-subject effect of response on intervention RT was also observed in the retrieval condition, with participants taking significantly longer to report unsuccessful retrieval than successful retrieval. However, this effect was not present in the study decision condition despite being observed in Experiment 2. The mixed results could be attributed to the inconsistent application of retrieval when participants were prompted to make study decisions rather than to retrieve.

Experiment 3 also demonstrated that reporting retrieval results took more time than making study decisions, even after ensuring the amount of the displayed instructions was balanced across both interventions. This suggests that the differences in reaction time (RT) between retrieval and study decision responses were attributable to the distinct cognitive processes underlying each intervention. Since retrieval was practiced during the retrieval intervention and was presumed to occur during the study decision intervention, the significant difference in response time supports the conclusion drawn from Experiment 2 that when retrieval is practiced as a monitoring tool during study decision-making, it appears to occur less frequently or at a shallower level compared to when learners are explicitly prompted to retrieve.

The results of Experiment 3 further replicated and extended the pattern observed in Experiment 2, showing that participants in the retrieval condition exhibited significantly higher global judgments of learning (JOLs) compared to other learning conditions (the single-study and study decision conditions). This finding, supported by a robust effect size (*η*^2^ = 0.16) and high statistical power, suggests that explicit retrieval prompts and practices may enhance learners’ confidence in their learning outcomes. Participants’ higher global JOLs in the retrieval condition likely reflect increased sensitivity to mnemonic cues such as retrieval fluency and the subjective ease of recall during active retrieval attempts. This metacognitive sensitivity might explain the observed overconfidence. Participants may overestimate their actual learning success when retrieval feels fluent or effortless, even if their actual memory performance is not enhanced.

## 5. General Discussion

The primary goal of the current study was to investigate whether learners monitor their learning using the strategy of retrieval when explicitly prompted to control their learning processes. The secondary goal of this study was to report an attempt to detect retrieval-based metacognitive monitoring using online and objective measures. Three experiments were conducted in pursuit of these two goals.

In Experiment 1, participants were prompted to regulate their learning processes by deciding whether to restudy an item or move on to the next one. The results showed that longer study decision response time was associated with higher recall accuracy, and study decision RT accounted for a significant proportion of variance in the final recall test performance. This suggests that retrieval may have been involved in the cognitive processes underlying study decision-making; a longer decision-making response time likely reflected greater retrieval effort, which facilitated better recall. However, study time was unexpectedly not associated with recall accuracy, indicating that extended study duration did not impact later retention. On the other hand, the low mean test accuracy (M = 0.16, SD = 0.13) raised concerns about a potential floor effect. To address this, Experiment 2 was conducted with a reduced number of learning targets to improve test performance.

Experiment 2 employed a between-subjects design with four groups (single-study, restudy, retrieval, and study decision) to better control total study time and directly compare the impact of two cognitive activities: retrieval and study decision-making. The similar behavior patterns observed in the study decision and retrieval groups, particularly in recall accuracy and response time by response, support our assumptions regarding the cognitive processes underlying study decision-making; participants tended to restudy items they struggled to retrieve and chose not to restudy those they successfully retrieved, suggesting retrieval was very likely engaged during the decision-making process. Additionally, the retrieval group spent significantly more time reporting their retrieval results than the study decision group did in making study decisions, indicating that retrieval may have been practiced less frequently or at a shallower level during study decision-making. Furthermore, Experiment 2 again found no effect of extended study time (12 s vs. 8 s) or prompts (retrieval and study decision prompts) on later retention, reinforcing the findings from Experiment 1.

Experiment 3 aimed to further investigate the differences in recall accuracy and intervention response time by response in both retrieval and study decision conditions as well as to examine the effects of prompts on recall accuracy using a within-subject design with three learning conditions: single-study, retrieval, and study decision. The significant differences in recall accuracy by response observed in both the retrieval and study decision conditions in Experiment 3 were consistent with those in Experiment 2, further supporting the assumed cognitive processes underlying the interventions. Specifically, restudy decisions were primarily driven by unsuccessful retrieval, whereas no-restudy decisions resulted from either successful retrieval or the absence of a retrieval attempt (as shown in Table 4). This pattern suggests that retrieval-based monitoring is very likely employed when learners are prompted to make study decisions. Notably, participants consistently spent more time reporting retrieval results than making study decisions. This finding suggests that retrieval occurs during study decision-making but at a lower frequency or shallower level, potentially involving a feeling of knowing rather than a complete recall, compared to when retrieval is explicitly prompted. This reduced cognitive engagement likely contributed to the shorter response times in the intervention of study decision-making than retrieval in both Experiment 2 and Experiment 3.

Moreover, Experiment 3 once again demonstrated no significant effect of the prompts on cued recall performance even after improving the recall tests accuracy. This unexpected yet consistently observed finding across all three experiments provides valuable insights. One possible explanation is that retrieval is inherently practiced during learning, regardless of the presence of prompts. Experiment 1 demonstrated that learners were aware of their retrieval practice when making study decisions. Similarly, in Experiment 3, most participants (74.32%) reported retrieving and rehearsing previous items when presented with new ones in the single-study condition. This suggests that learners actively and consciously retrieve and rehearse previous items during learning, even when new items are introduced, highlighting the spontaneous nature of metacognitive monitoring. This spontaneous retrieval and rehearsal may explain the lack of prompt effects in Experiments 2 and 3, as the learners’ intrinsic retrieval processes likely overshadowed any external prompts. These findings suggest that retrieval is an integral component of self-regulated learning, occurring naturally without the need for explicit prompting.

The current findings highlight the engagement of retrieval-based monitoring as a metacognitive tool guiding self-regulated learning decisions. These findings have potential implications for intelligence research, particularly in how individuals monitor and regulate their own learning. Both the metacognition (Dunlosky and Metcalfe 2008; Nelson and Narens 1990; Thiede et al. 2003) and self-regulated learning frameworks (Koriat 2007; Nelson and Leonesio 1988; Winne and Hadwin 1998) commonly propose that monitoring guides control processes, which in turn affect learning outcomes. Learners who engage in retrieval to assess their understanding of the material are more likely to better monitor their learning process and thereafter show better performance on later tests compared to those who do not. This suggests that individuals who habitually use retrieval as a metacognitive strategy may also perform better on other cognitive tasks, supporting the notion that effective monitoring and self-regulated learning are linked to broader cognitive abilities.

From a practical perspective, this finding could inform future instructional design, particularly in computer-assisted learning, by indicating that prompting retrieval through additional instructions may not always be necessary, thereby streamlining the learning process. Theoretically, understanding learners’ spontaneous metacognitive monitoring has significant implications for self-regulated learning. While previous research has documented evidence of spontaneous monitoring, particularly in the form of self-testing (e.g., Bottiroli et al. 2010), future research should further explore the hypothesis that retrieval-based metacognitive monitoring is spontaneously practiced in self-regulated learning to deepen our understanding of its role and characteristics in cognitive processes.

In conclusion, all three experiments consistently demonstrate that retrieval is engaged in the cognitive processes underlying study decision-making when learners are prompted to make such decisions. The results further suggest that retrieval occurs less frequently or at a shallower level when learners are prompted to make study decisions compared to when they are explicitly prompted to retrieve. These findings indicate that learners employ retrieval-based monitoring to regulate their learning, but its application is inconsistent. The present analysis, primarily based on behavioral measures such as decision-making response time and final test accuracy, highlights the potential for using objective, online methods to detect retrieval-based metacognitive monitoring. This suggests promising avenues for future research in developing more objective and automated techniques to track learners’ cognitive strategies during self-regulated learning.

## 6. Limitations and Future Directions

The study has several limitations that should be considered. Firstly, the effect of the study decision prompt remains uncertain. While the prompt was designed solely to encourage learning control, it may have been inadvertently perceived as a monitoring prompt, thereby triggering metacognitive monitoring. As a result, it is possible that the study decision prompt not only facilitated study control but also influenced participants’ monitoring processes.

Secondly, in all three experiments, a distractor task, a two-digit addition question, was posted to prevent participants from rehearsing the target items in working memory. The average response time for the distractor task ranged from 6 to 7 s across experiments. According to Goldstein (2008), information in working memory typically decays in 10–15 s unless it is actively attended to. Since the mean distractor task response time was shorter than this decay window, the target items may have remained active in working memory during the study decision process, potentially reducing the need for retrieval. Future studies should test this argument with distractor tasks that occupy participants’ attention for a longer duration.

Thirdly, metacognitive monitoring in this study was assessed indirectly through behavioral measures, specifically study decision response time and final recall accuracy. While these measures offer objective and online insight into cognitive processes, they are not direct indicators of monitoring and rely on interpretative analysis. As proposed by Veenman (2005), eye-tracking technology has the potential to provide more direct, objective, and online evidence of cognitive and metacognitive processes. Future studies may consider integrating eye-tracking and electroencephalogram (EEG) technology to gain a more direct and precise understanding of monitoring mechanisms.

Another notable limitation of the present study is the relatively small sample size in each experiment, which likely contributed to the low to medium statistical power observed in post hoc analyses. While the consistency of findings across three experiments lends support to the observed patterns, future studies with larger and more adequately powered samples are needed to confirm the reliability and generalizability of these results.

Additionally, this study focused on university students, which may limit the generalizability of the findings to individuals of different age groups or educational backgrounds. However, this sample was intentionally selected for its theoretical and practical relevance to the research questions. University students are a population that routinely engages in self-regulated learning (SRL) tasks, making them an ideal group for investigating the role of retrieval-based metacognitive monitoring in the process of study decision-making (e.g., time allocation, restudying choices). Their cognitive maturity and frequent exposure to metacognitive demands in academic settings also provide a high need to understand the study decision-making process in self-regulated learning. Moreover, the results are particularly applicable to higher education contexts, where understanding the role of retrieval-based metacognitive monitoring played in study decisions and control can directly inform teaching practices and learning interventions.

Furthermore, the current experiments did not measure individual differences in cognitive abilities (e.g., working memory capacity, executive functions) that might influence how learners engage retrieval during study decision-making in self-regulated learning. Future studies could explore whether individuals with higher cognitive abilities would rely more heavily on retrieval when making study decisions and achieve higher memory performance.

A further limitation of this study concerns the analysis of single-item measures such as reaction times, binary accuracy scores, and study decisions. This study mostly analyzed single-item measures by averaging them per participant, which does not fully account for the nested structure of items within individuals. Future research should use multilevel models to better capture both item- and participant-level variability.

## 7. Conclusions

The present study provides important insights into the engagement of retrieval during study decision-making in self-regulated learning. Our findings suggest that learners spontaneously engage in retrieval-based monitoring during learning, even in the absence of explicit prompts. When prompted to make study decisions, learners appear to engage in retrieval at a lower frequency and shallower level compared to when explicitly cued to retrieve information. This spontaneous retrieval likely plays a crucial role in monitoring their learning processes, influencing subsequent study decisions and recall accuracy. Furthermore, the results indicate that prompting retrieval or study decisions does not significantly improve retention, likely due to learners’ intrinsic metacognitive practices. The study also explored the detection of retrieval-based metacognitive monitoring using objective and online measures, specifically using response time and final recall accuracy.

Overall, this study contributes to our understanding of metacognitive monitoring in learning contexts and offers valuable implications for the design of educational interventions and learning technologies. By recognizing the natural occurrence of retrieval-based monitoring, educators and instructional designers may reconsider the necessity of additional prompting, making learning processes more efficient and in line with learners’ inherent cognitive strategies.

## Figures and Tables

**Table 1:** Descriptive statistics for study decision measures in Experiment 1: means, standard deviations, skewness, and kurtosis.

	*n*	M	SD	Skewness (SE)	Kurtosis (SE)
Study decision response	39	0.79	0.26	−1.39 (0.38)	1.74 (0.74)
Study decision RT (ms)	39	1118.82	596.69	0.93 (0.38)	0.45 (0.74)
Test accuracy	39	0.16	0.13	1.07 (0.38)	0.80 (0.74)
Global JOL	38	25.34	15.02	0.57 (0.38)	−0.53 (0.75)
Individual JOC	39	17.78	13.82	1.00 (0.38)	0.93 (0.74)

**Table 2:** Correlations between variables in Experiment 1.

Variables	1	2	3	4	5
1. Study decision	-	−0.33 *	−0.13	0.03	−0.17
2. Study decision RT		-	0.34 *	0.34 *	0.37 *
3. Test accuracy			-	0.55 ***	0.71 ***
4. Global JOL				-	0.45 **
5. Individual JOC					-

* *p* < 0.05; ** *p* < 0.01; *** *p* < 0.001.

**Table 3:** Results of the generalized linear mixed model assessing the impact of item-level study decisions and item-level study decision response time (RT) on item accuracy in Experiment 1.

Model	Logit
Fixed part	Coeff. (s.e.)
Intercept	−2.01 (0.17)
Item-level study decision	−0.02 (0.15)
Item-level study decision RT	0.13 (0.04)
Random part	
Participant	1.20 (1.09)
Swahili–English word pair	1.08 (1.04)
Deviance (AIC)	13,761.58

**Table 4:** Regression statistics.

	B (SE)	95% CI	*t*	*df*	*p*
Contant	1058.60 (26.89)	[1005.82, 1111.38]	39.36	2728	<0.001
Test accuracy	367.80 (66.46)	[237.44, 498.16]	5.33	2728	<0.001

**Table 5:** Assumptions of the cognitive processing for the responses in the retrieval and study decision group in Experiment 2.

Group	Responses	Assumed Cognitive Processes	
		Attempt to Retrieve	Success of Retrieval
Retrieval	Remember	+	+
	Not remember	+	−
Study decision	Next	+	+
		−	N/A
	Restudy	+	−

Note: “+” refers to the existence of that cognitive processing; “−” refers to the absence.

**Table 6:** Means, standard deviations, skewness, and kurtosis of test accuracies and global JOLs in all groups in Experiment 2.

Group		Test Accuracy				Global JOL (%)			
	*n*	M	SD	Skewness (SE)	Kurtosis (SE)	M	SD	Skewness (SE)	Kurtosis (SE)
Single-study	22	0.16	0.14	1.17 (0.49)	0.61 (0.95)	31.95	23.91	0.21 (0.49)	−1.39 (0.95)
Restudy	17	0.16	0.11	1.16 (0.55)	2.89 (1.06)	32.82	14.62	−0.34 (0.55)	−0.43 (1.06)
Retrieval	17	0.21	0.15	1.14 (0.55)	1.37 (1.06)	45.06	23.20	−0.08 (0.55)	−0.71 (1.06)
Study decision	18	0.17	0.15	1.35 (0.54)	1.30 (1.03)	39.44	19.24	0.03 (0.54)	−1.10 (1.04)

**Table 7:** Means, standard deviations, skewness, and kurtosis of test accuracies and global JOLs in Experiment 3.

Condition	Test Accuracy					Global JOL (%)				
	*n*	*M*	*SD*	Skewness (SE)	Kurtosis (SE)	*n*	*M*	*SD*	Skewness (SE)	Kurtosis (SE)
Single-study	74	0.37	0.21	0.73 (0.28)	0.25 (0.55)	71	36.00	19.36	0.07 (0.29)	−0.58 (0.56)
Retrieval	74	0.39	0.23	0.23 (0.56)	−0.37 (0.55)	66	46.71	20.54	−0.27 (0.30)	−0.42 (0.58)
Study decision	74	0.36	0.23	0.90 (0.28)	0.77 (0.55)	71	36.38	21.21	0.31 (0.29)	−0.74 (0.56)

## Data Availability

Data are available on the Open Science Framework: https://osf.io/r3c9q/?view_only=585aea2db1984360a3dbc4fb8d179420, accessed on 5 August 2025.

## Note

1 In order to eliminate ordering effects and systematic bias, a between-subjects ANOVA was conducted to compare the test accuracies of only the first conditions completed by each participant to ensure no prior exposure to other conditions could influence results. The analysis showed no significant differences in test accuracy between the initial learning conditions, *F*(2, 71) = 0.52, *p* = 0.60, *η*2 = 0.02 (single-study learning condition, *M* = 0.31, *SD* = 0.19, *n =* 29; retrieval learning condition, *M* = 0.26, *SD* = 0.19, *n =* 24; study-decision learning condition, *M* = 0.30, *SD* = 0.23, *n =* 21).
